# Thermal conditions during early life influence seasonal maternal strategies in the three-spined stickleback

**DOI:** 10.1186/s12898-017-0144-x

**Published:** 2017-11-10

**Authors:** Sin-Yeon Kim, Neil B. Metcalfe, Alberto da Silva, Alberto Velando

**Affiliations:** 1 Departamento de Ecoloxía e Bioloxía Animal, Universidade de Vigo, 36310 Vigo, Spain; 2 Institute of Biodiversity, Animal Health and Comparative Medicine, College of Medical, Veterinary and Life Sciences, University of Glasgow, Glasgow, G12 8QQ UK

**Keywords:** Carotenoid, Climate change, Life-history, Maternal effect, Phenotypic plasticity

## Abstract

**Background:**

Conditions experienced by a female during early life may affect her reproductive strategies and maternal investment later in life. This effect of early environmental conditions is a potentially important mechanism by which animals can compensate for the negative impacts of climate change. In this study, we experimentally tested whether three-spined sticklebacks (*Gasterosteus aculeatus*) change their maternal strategy according to environmental temperatures experienced earlier in life. We studied maternal investment from a life-history perspective because females are expected to adjust their reproductive strategy in relation to their current and future reproductive returns as well as offspring fitness.

**Results:**

F1 families were reared in control and elevated winter temperatures and their reproductive trajectories were studied when returned to common conditions. Females that had experienced the warm winter treatment (n = 141) had a lower fecundity and reduced breeding and total lifespan compared to the control individuals (n = 159). Whereas the control females tended to produce their heaviest and largest clutches in their first reproductive attempt, the warm-acclimated females invested less in their first clutch, but then produced increasingly heavy clutches over the course of the breeding season. Egg mass increased with clutch number at a similar rate in the two groups. The warm-acclimated females increased the investment of carotenoids in the first and last clutches of the season. Thus, any transgenerational effects of the maternal thermal environment on offspring phenotype may be mediated by the allocation of antioxidants into eggs but not by egg size.

**Conclusions:**

Our results indicate that conditions experienced by females during juvenile life have a profound effect on life-time maternal reproductive strategies. The temperature-induced changes in maternal strategy may be due to constraints imposed by the higher energetic costs of a warm environment, but it is possible that they allow the offspring to compensate for higher energetic costs and damage when they face the same thermal stress as did their mothers.

**Electronic supplementary material:**

The online version of this article (10.1186/s12898-017-0144-x) contains supplementary material, which is available to authorized users.

## Background

There is increasing evidence that conditions experienced by a female can influence her offspring’s phenotype via non-genetic maternal effects [1]. Environmentally-induced maternal effects typically include maternal oviposition decisions [2], maternal care [3] and egg size [4]. Active or passive transmission of maternal trace elements or hormones to eggs or embryos may provide another mechanism to adjust female strategy to environmental conditions [5, 6].

Changes in reproductive strategies and maternal investment of females in response to the environment must be addressed from a life-history perspective because adaptive maternal effects for offspring can come at a cost to females [6]. Increased investment of resources and energy in eggs and altered timing of reproduction entail direct costs for the female and will reduce her future reproductive success [7]. Other maternal effects, such as transfer of hormones to eggs or embryos, may incur indirect costs due to negative pleiotropic effects of increased hormone levels on the physiology or behaviour of the mother [8]. Thus, females are expected to adjust their maternal strategy in relation to their current and future reproductive returns as well as offspring fitness. Indeed, it has been shown in soil mites (*Sancassania berlesei*) that egg and clutch size shift dynamically with female age, creating asymmetry in the competitive abilities of older and younger offspring from the same mother [9]. This suggests that the significance of female strategies may be misinterpreted when studies consider only a snapshot of maternal effects, based on a single reproductive attempt.

Increased temperatures due to current climate change often have negative impacts on fitness of ectotherms whose body temperatures conform to environmental temperatures, particularly in populations that live close to the species’ thermal maximum [10–12]. In ectotherms, a higher temperature leads to a higher resting metabolic rate [13], and so adjustments to oxygen delivery or oxidative damage may be critical in coping with thermal stress [14–17]. Increased temperatures also have a negative effect on the body size of aquatic ectotherms from the individual to community structure levels [18]. Since metabolic rate increases as a function of body size as well as environmental temperature [19], it is expected that increased temperatures should lead to a decrease in the mean body size if the supply of resources does not increase in parallel [20]. Transgenerational plasticity is a potentially important mechanism by which animals can compensate for negative impacts of thermal stress. Indeed, some previous studies on fish species have shown that parental exposure to thermal stress can induce changes in offspring phenotype [21–23]. Adaptive maternal strategies in response to increased temperatures are expected to alter physiological or life-history phenotypes of offspring, for example by enhancing stress resistance or reducing body size. In fishes, maternal antioxidants deposited in eggs, such as carotenoids and vitamin E, provide protection for offspring against deleterious effects of free radicals produced during normal metabolism and growth [24, 25]. However, it is unknown whether females can adjust maternal transfer of antioxidants to eggs in response to expected thermal conditions for their offspring.

In this study, we experimentally tested whether female three-spined sticklebacks (*Gasterosteus aculeatus*), originating from a population close to the low-latitude margin of the species’ distribution (and hence at risk of exposure to increasingly stressful environmental temperatures), change their seasonal reproductive strategies in response to increased water temperatures experienced during juvenile life. In this annual population, females spawn more frequently throughout a single relatively long breeding season compared to more northern populations [26, 27]. F1 full-sib families were reared under normal and elevated winter temperatures prior to the reproductive season. Our previous studies have shown that this manipulation of winter temperature influenced the reproductive strategy of males, altering the seasonal pattern of carotenoid investment in nuptial colouration [27] and the expression of genes related to metabolic processes and oxidation reduction [28]. In the three-spined stickleback, maternal transfer of carotenoids to eggs can trade off with other critical functions such as somatic maintenance or sexual selection [29]. Here, we examined whether exposure to warmer winter temperatures prior to the breeding season altered the seasonal patterns of investment by females in clutch mass, clutch size, egg mass and egg carotenoids, and so tested whether females adjust maternal strategies according to the expected environmental conditions. Since individual variation in the expectation for future reproduction is an important determinant of reproductive trajectories, we also tested how overall productivity interacted with seasonal variation in maternal investment under the contrasting thermal regimes. We predicted that, in general, females would reduce egg size but increase egg carotenoids when they experience warmer temperatures because small body size and maternal antioxidants can allow offspring to reduce their metabolic costs and oxidative damage under warm developmental conditions. The temperature treatment was also expected to influence the body condition of the females, thereby modulating the seasonal patterns of their maternal strategies.

## Methods

### Study population, fish husbandry and temperature manipulation

Wild three-spined sticklebacks captured in the Rio Ulla, Spain were crossed in spring 2013 to obtain full-sib families (for details, see [27, 30]). Randomly selected F1 individuals from 32 full-sib families were reared in 8-L growth tanks (n = 114 tanks), each initially housing 11 or 12 unsexed juveniles, with either two or four such tanks per family. Two fish per tank were subsequently removed at age 5 months to be used in another experiment [31]. The growth tanks were connected to four closed water systems, in which water was continuously filtered, aerated and temperature-controlled by the combined flow-through function. Fish were fed to satiation once a day on a progressive diet of newly hatched *Artemia* until age 3 months and a commercial pelleted diet from age 2 months onwards (Gemma Micro, Skretting, Norway). The dry food pellets contained a high level of carotenoids (103.9 µg g^−1^, [32]). The natural photoperiod for the source population was simulated by programmed LED illumination, reaching a maximum of 15 h of day length in June and a minimum of 9 h in December; similarly the water temperature in the growth tanks reflected the natural pattern, reaching a peak of 20 °C in August then falling steadily until the experimental manipulation of winter temperatures began.

At 6 months of age juveniles were permanently marked with colour elastomer tags (Northwest Marine Technologies, Shaw Island, WA, USA) under a low dose of benzocaine anaesthetic to track individual life-histories (n = 1038 individuals). At this point (November) half of the growth tanks were assigned to the control and the other half to the warm winter treatment, with approximately equal numbers of fish in each family in each treatment. Water temperature in the control group was gradually reduced from 14 °C in November to 9 °C in January then increased to 14 °C in March to maintain the simulation of the ambient temperature regime for the source population, whereas the temperature in the experimental group was maintained at 14 °C during this period, so that the experimental fish experienced warmer winter temperatures than the control fish (see also [27]). Randomly selected samples from the two treatment groups were sacrificed with an overdose of benzocaine anaesthetic during the experiment to explore genetic mechanisms of life-history plasticity in a parallel study (n = 41 control and 46 warm-treated individuals at age 7 months; n = 29 control and 27 warm-treated individuals at age 10 months; n = 58 control and 53 warm-treated individuals at age 13 months).

### Female life-histories

The standard length of all F1 individuals was measured at the onset of the breeding season (4–7 February 2014). Females were kept in their growth tanks and monitored daily to record when they became gravid (evident from a swollen abdomen). A subsample of sexually mature males was moved to individual tanks for a parallel study [27], leaving one or two males per growth tank together with females during the reproductive season. A total of 159 control and 141 experimental females, excluding the sacrificed samples, spawned between February and August 2014, producing 1891 clutches. Whenever a female became fully gravid, the egg clutch was stripped by applying gentle pressure to the abdomen under light benzocaine anaesthetic. The clutch was weighed and gently spread on a piece of blotting paper using a fine painting brush then photographed, for later determination of clutch size. In this way, the eggs were handled for less than 1 min and excess water was removed before eggs were stored at – 80 °C for later carotenoid analysis. On 329 occasions out of 1891, gravid females spawned in their tank before we could strip and photograph the eggs, but the spawning events were recorded. By the end of August all females in the growth tanks had ceased egg production, and so we stopped monitoring females. Since the clutch size was highly repeatable within individuals (see “Results”), fecundity was estimated as the mean known clutch size multiplied by the number of spawning episodes. This could not be calculated in 10 individuals that produced only one clutch, which was of unknown size. Reproductive lifespan was calculated as the time (days) elapsed between the date on which a female’s first and last spawning occurred.

### Analysis of egg carotenoids

We extracted carotenoids from the collected clutch samples and determined the total carotenoid concentration following JD Blount, NB Metcalfe, TR Birkhead and PF Surai [33]. A similar subsample of eggs, approximately 20 mg, was taken from each clutch sample and weighed to the nearest 0.01 mg. The subsamples were homogenized with 250 µl mixture (1:1) of 5% sodium chloride and ethanol, and then carotenoids were extracted twice using 375 µl *n*-hexane. After each extraction, the subsample was centrifuged, and then the hexane, containing the carotenoids, was collected and evaporated. The extracted carotenoids were dissolved in 125 µl ethanol to determine the total carotenoid concentration in a spectrophotometer (Synergy HT, BioTek, Winooski, VT, USA). Carotenoids were identified as a single peak at 440 nm, and concentrations (expressed as µg g^−1^) were determined using a lutein curve as a standard.

### Statistical analyses

We tested whether life-history traits differed between females from the control and warm winter groups. Size at maturation (standard length at the beginning of the breeding season), date of first spawning, reproductive lifespan (time between the first and last spawnings), number of spawning events and estimated fecundity were analysed as dependent variables in linear mixed-effect (LME) models. Where the frequency distribution of dependent variables differed from normal, these were transformed prior to the analyses (size at maturation: one sample Kolmogorov–Smirnov test: Z = 0.082, p < 0.001, ln-transformed; number of spawning events: Z = 0.090, p < 0.001, square root-transformed; estimated fecundity: Z = 0.080, p < 0.001, square root-transformed). Models with the transformed variables satisfied the linearity and homoscedasticity criteria. In each LME model, experimental treatment was included as a fixed factor (independent variable) and growth tank and full-sib family as nested random effects. In the analysis of estimated fecundity, body length at maturation was also included as a covariate [full model: estimated fecundity = treatment + body length, random effect: growth tank (full-sib family)]. The survival of females during the breeding season was analysed by using a Cox proportional hazard model, including treatment as a fixed effect and full-sib family identity as a random effect.

Although ageing is clearly defined as any age-specific pattern in variables associated with individual fitness, it is often difficult to decide how to quantify life-history trajectories in different animals. In annual fish populations like our study population, females produce multiple clutches throughout a single breeding season. To examine ageing patterns in reproductive lifespan and spawning trajectories of the control and experimental fishes, we first explored how the probability of a spawning event being the last (= terminal) one was related to clutch number (i.e. how many clutches the female had produced at that time). We created a binary variable, categorizing each breeding attempt of a female as the last spawning event of the season (1) or not (0), and analysed it using a generalized linear mixed model (GLMM) with a binomial error structure and logit link function. Experimental treatment (control or warm winter), clutch number (CN) and its quadratic term and their interactions were included as fixed effects in the model. In this model, female identity, growth tank and full-sib family were included as nested random effects. We also explored inter-clutch interval (measured in days) to examine whether control and experimental fish produced clutches at different rates.

Since inter-clutch interval did not significantly differ between the control and experimental females (see “Results”), we used clutch-specific traits [i.e. clutch mass, clutch size, egg mass (i.e. clutch mass/clutch size) and egg carotenoid concentration] to analyse longitudinal life-history patterns according to the clutch number. In a LME model fitted to repeated measures of each clutch-specific trait, experimental treatment, total number of spawning events (NS) and clutch number (CN) were included as fixed effects. Since reproductive performances across reproductive events can change in a non-linear form [27], the quadratic term of clutch number (CN^2^) was additionally included in the model where significant. Significant two- and three-way interactions were also included in the model. In all LME models female identity, growth tank and full-sib family were included as nested effects on random intercepts and slopes (clutch number across individuals). All statistical analyses were performed using the *glmer* function of the lme4 package or the *lme* function of the nlme package in R 3.3.1.

## Results

### Effects of winter temperature on body size, fecundity and lifespan of females

**Fig. 1:**
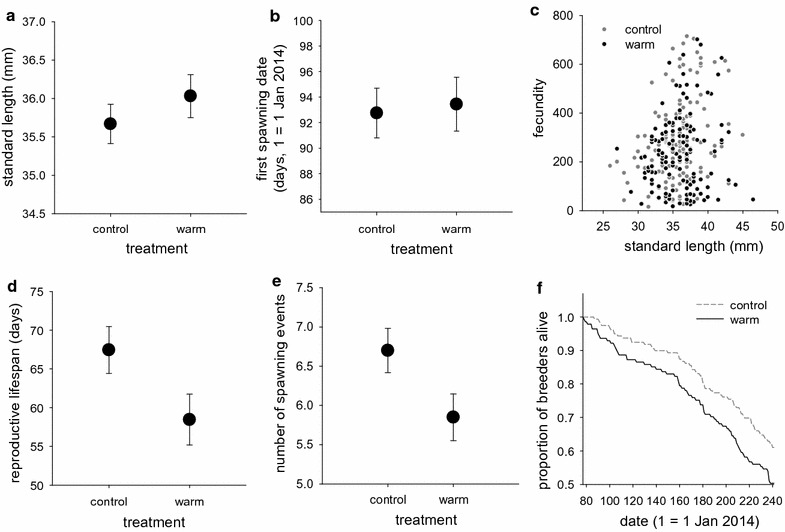
Comparisons of key life-history traits of female three-spined sticklebacks between the control (n = 159 females) and warm winter (n = 141 females) groups. Mean ± SE **a** standard length at the onset of the breeding season, **b** date of first spawning, **c** relationship between standard length and fecundity (total number of eggs produced during the breeding season), **d** reproductive lifespan calculated as date at last spawning – date at first spawning, **e** total number of spawning events and **f** survival curves for females during the reproductive season

Neither body size (standard length) at the beginning of the reproductive season nor first spawning date differed significantly between the control and warm-treated females (Fig. 1a, b; see also Additional file 1: Figure S1a for transformed data; LME: standard length: F_1,76_ = 0.800, p = 0.373; first spawning date: F_1,76_ = 0.024, p = 0.877). However, the warm-treated females produced significantly fewer eggs than the controls over the entire breeding season (Fig. 1c; see also Additional file 1: Figure S1b; F_1,75_ = 7.216, p = 0.009) when a positive correlation between body size and egg production was taken into account (Fig. 1c; F_1,181_ = 10.638, p = 0.001). This was partly because the warm-treated females had a tendency for a shorter reproductive lifespan (Fig. 1d; F_1,76_ = 3.873, p = 0.053) and fewer spawning events (Fig. 1e; see also Additional file 1: Figure S1c; F_1,76_ = 5.657, p = 0.020). In addition, analysis of survival during the season revealed that the warm-treated female breeders had a shorter lifespan than the control females (Fig. 1f; Cox proportional hazard model: β ± SE = 0.448 ± 0.180, $\chi_{1}^{2}$ = 6.17, p = 0.013).

**Fig. 2:**
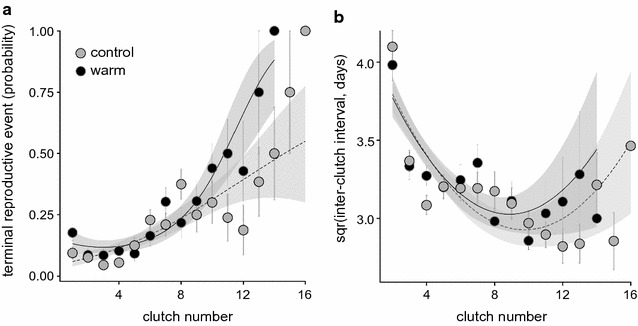
Change across clutches in **a** the probability of being the terminal spawning event and **b** inter-clutch interval with respect to the winter temperature treatment (control or warm winter). Clutch-specific mean ± SE are presented, and fitted lines are derived relationships and 95% CI in GLMM and LME models

Analysis of the probability of a spawning event being the terminal clutch showed that there was significant interacting effect of treatment and quadratic clutch number (GLMM: treatment × CN^2^, $\chi_{1}^{2}$ = 10.76, p = 0.001). The probability of the first spawning being the terminal one was higher for the warm-treated females (Fig. 2a; 25 out of 141 warm water individuals produced a single clutch, in comparison to only 15 out of 159 control individuals; Pearson’s Chi square test: $\chi_{1}^{2}$ = 4.451, p = 0.035). The probability of a spawning being the last clutch of the season also rose more steeply from the 10th clutch onwards in the warm-winter females than in controls (Fig. 2a).

### Effects of winter temperature on seasonal patterns of reproductive investment

Among the females that produced more than one clutch, the overall mean and trajectory of inter-clutch interval (latency between two consecutive spawning events) did not differ between the control and warm winter groups but changed over time (Fig. 2b; LME: effect of treatment: F_1,70_ = 1.354, p = 0.249; CN: F_1,1327_ = 87.33, p < 0.001; CN^2^: F_1,1327_ = 48.05, p < 0.001; treatment × CN: F_1,1327_ = 2.632, p = 0.105; treatment × CN^2^: F_1,1327_ = 0.203, p = 0.652). The interval between the first and second clutches was the longest in both treatment groups (an average of 17.6 days), but this interval then declined and remained relatively stable over the rest of the season (average interval between subsequent clutches being only 10.7 days).

**Fig. 3:**
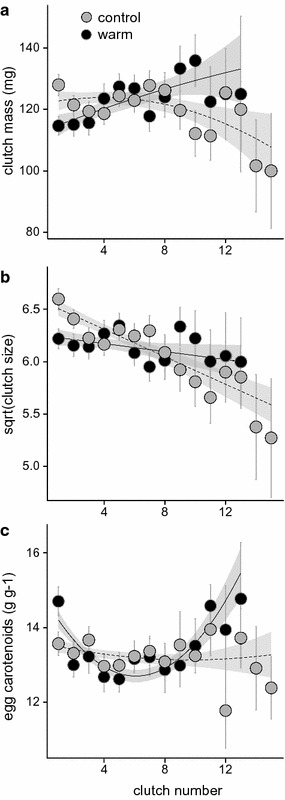
Change across clutches in **a** clutch mass, **b** clutch size and **c** egg carotenoid concentration with respect to the winter temperature treatment (control or warm winter). Clutch-specific mean ± SE are presented, and fitted lines are derived relationships and 95% CI in GLMM and LME models

Seasonal changes in clutch mass were affected by winter temperature treatment (treatment × CN: F_1,1290_ = 14.05, p < 0.001; for full results, see Additional file 1: Table S1). The control females showed a continuous decline in clutch mass over the breeding season, whereas the warm-treated females initially produced relatively light clutches but then increased their clutch mass over the season (Fig. 3a). These seasonal trends were affected by an interaction between number of spawnings and clutch number (NS × CN: F_1,1290_ = 12.43, p < 0.001; Additional file 1: Table S1), with the females producing the most clutches showing a delayed decline in clutch mass compared with those producing the fewest (Additional file 1: Figure S2a), but this pattern was similar in the two treatments (non-significant treatment × NS × CN and treatment × NS × CN^2^ interactions). The equivalent analysis of clutch size also showed a significant interaction between treatment and clutch number (F_2,1290_ = 13.29, p < 0.001; Additional file 1: Table S2) but no interacting effect of number of spawnings. The control females showed a continuous decline in clutch size across consecutive spawnings, whereas the experimental individuals started producing relatively small clutches at the beginning of the season but then were more able to maintain clutch size as the season progressed (Fig. 3b).

Seasonal changes in egg mass did not differ between the control and warm-treated females (Additional file 1: Table S3) but there was a significant interacting effect of clutch number and number of spawnings (NS × CN: F_1,1289_ = 5.467, p = 0.019, Additional file 1: Table S3). In general, females produced larger eggs in later clutches (Additional file 1: Figure S2b). Individuals producing intermediate numbers of clutches produced the largest eggs, while females producing fewest clutches also produced the smallest eggs, especially at the beginning of their breeding season (Additional file 1: Figure S2b).

The seasonal pattern of egg carotenoid concentration was affected by winter temperature treatment (Treatment × CN^2^: F_1,1075_ = 7.420, p = 0.007; Additional file 1: Table S4). Although the overall level of egg carotenoids did not differ between the two treatment groups, the warm-treated females produced eggs with elevated carotenoid concentration at the beginning and at the end of their reproductive life, whereas the control females produced eggs with a similar level of carotenoids throughout the season (Fig. 3c).

## Discussion

Our study demonstrates that female sticklebacks adjust their seasonal patterns of reproductive investment in response to environmental conditions that they experienced earlier in life. Warmer winter temperatures prior to the breeding season did not influence either their body size at maturation or their timing of first reproduction. However, the warm-acclimated females had a lower fecundity and reduced lifespan compared to those reared at normal winter temperatures. The warm-acclimated and control females spawned at similar intervals within the season, but their reproductive investment trajectories in clutch mass, clutch size and concentration of egg carotenoids differed.

In contrast to the response by females, male sticklebacks in the same warm winter temperature treatment (i.e. the brothers of the females analysed in the present paper) showed a clear plasticity in body size and timing of reproduction (i.e. expression of red nuptial colouration): the warm-acclimated males were smaller at the beginning of the breeding season [28] but started to express red colouration earlier than the control males [27]. Female sticklebacks begin mobilizing energy into ovarian and support tissue in winter then continue to grow throughout the breeding season, whereas males stop growing at the onset of maturation [34, 35]. Thus, contrasting energy allocation strategies could explain the different life-history plasticity between the two sexes. In ectotherms, the approximately exponential effects of temperature on energy expenditure are well established. These effects are partly due to direct influences on the kinetic energy of cellular components [19, 36]. But while energetic costs increase exponentially with increasing temperature, food intake only increases up to a peak, above which food intake declines steeply with further increases in temperature [37]. Feeding and digestion rates of fish in a warming environment are thus metabolically constrained even if food is freely available [38]. Food was provided only once a day to both control and warm-acclimated individuals, so the possibility that fish increased feeding to compensate for the metabolic costs of living in a warm environment was probably none or minor. Thus, energy for somatic growth and maintenance was probably limited during winter in both control and warm environments. It is possible that the warm-acclimated females compensated for the increased energetic costs of living in warmer water by reducing their allocation of energy to reproductive functions during the winter (while maintaining their investment in somatic growth) [19, 36]. In accordance with this hypothesis, the warm-acclimated females produced fewer eggs (primarily because they laid fewer clutches), terminated reproduction earlier and suffered higher mortality during the season than the control females, probably due to increased energetic costs for somatic maintenance and growth [39]. Warmer conditions during the winter may have promoted physiological changes in metabolic rate and cardiac function in a manner that incurred higher energetic costs to the fish [23, 40]. It is also possible that higher temperatures either caused more oxidative damage or reduced the amount of resources available to the fish to repair damage [41].

The warm-acclimation not only reduced the overall fecundity and lifespan of females but also altered their strategic allocation of resources among reproductive attempts. In iteroparous organisms the cost of reproduction leads to a trade-off between reproductive effort at any given time and expectation of future reproduction [42, 43]. Thus, life-history theories predict that breeders will optimize the temporal pattern of reproductive investment according to both their life expectancy and the time-dependent fitness gain from a given reproductive attempt [44–46]. Females reared in the control environment invested relatively more in their first spawning, producing heavier and larger clutches, compared to their later spawning events, whereas the warm-treated females produced increasingly heavier clutches with a similar number of eggs across clutches. This suggests that thermal conditions during early life can shape a female’s strategy for allocating energy and resources between early and late reproductive attempts. The warm-acclimated females may have had fewer resources available for initial reproductive investment than the control females due to their higher energetic costs prior to the breeding season. They then invested more in reproduction as the season progressed probably because their prospects for survival and reproduction were lower than for the control females.

An alternative explanation is that the differential allocation strategies of females reflect their expectation of the conditions that their offspring will encounter during growth. This life-history plasticity can be adaptive if fitness benefits of reproducing early in the season [47, 48] are greater in colder or more seasonal environments. Fish born late in the season may incur a greater risk of overwinter mortality (due to small size) and/or a reduced likelihood of attaining a size that allows reproduction the following year [49, 50], but these handicaps may be reduced for juvenile fish that live in environments with warm winters. Thus, the warm-treated females may benefit by reducing their investment in early reproduction and so delaying reproductive senescence. In accordance with this observation, our previous study has shown that male sticklebacks grown in a warmer environment also subsequently reduced their investment in costly sexual signals early in the breeding season, and consequently senesced at a slower rate [27].

Our results show that any transgenerational effects of the maternal thermal environment on offspring phenotype may be mediated by the allocation of antioxidants into eggs but not by egg size. A previous study of marine sticklebacks also found that female acclimation temperature did not influence egg size [23], and in general egg size in sticklebacks shows less phenotypic plasticity than do other reproductive traits such as the number of spawnings [51]. In our study, egg mass increased with clutch number at a similar rate in the control and warm-treated females. Since the breeding season in this annual fish population is extremely long, producing larger eggs in the later clutches may be an adaptive strategy for a female to compensate for the competitive disadvantages and shorter growing season of late-hatching offspring. In fish, larvae hatching from larger eggs are more likely to survive than those hatching from smaller ones, but there is no general evolutionary trend towards greater egg size [52]. Because juvenile fish that attain a larger size at the onset of winter suffer less overwinter mortality [53], selection may favour the female strategy of producing larger eggs in the later clutches. On the other hand, we found evidence that female sticklebacks modulate maternal transfer of antioxidants to eggs according to the predicted thermal environment of the offspring. In the control females, the concentration of egg carotenoids was maintained at a similar level across subsequent clutches despite the senescent decline in clutch size and increase in individual egg mass. However, the females reared in warmer temperatures increased the investment of these maternal antioxidants in the first and last clutches of the season.

Carotenoids are important for reducing oxidative stress and provide the pigment for colour signals used in sexual selection in many bird and fish species [33, 54, 55]. Some experimental studies of birds have shown that maternal carotenoids deposited into eggs can have profound effects on offspring fitness by enhancing their survival during embryonic and nestling periods and the eventual ornamental coloration as adults [56, 57]. In fishes, egg carotenoids positively influence survival and disease resistance of larvae during development and juvenile body size during growth ([25, 58], but see [59, 60]). Carotenoids transferred from the mother may enhance the physiological condition of offspring during their development and growth, for example, by affecting the expression of phenotypic variation in antioxidant capacity or endocrinal signal [61]. Such phenotypic programming by mothers may be important for offspring to cope with increased oxidative stress due to greater rates of metabolism in the predicted warm environment [13]. Transgenerational effects of the maternal environment on metabolic or antioxidant capacities should shape thermal reaction norms of offspring traits in a warming environment [23]. However, it is unclear why in our study the warm-acclimated female sticklebacks increased the investment of maternal antioxidants only in their earliest and last clutches. Females may be able to adjust their investment of carotenoids between egg composition, influencing offspring phenotypic variation among clutches, and their personal use for self-maintenance during the reproductive season [29, 62]. It is possible that the warm-acclimated females tended to invest more heavily in their last clutch (i.e. terminal investment) because they had a reduced life expectancy than those reared at normal winter temperatures [46, 63].

## Conclusions

In general, elevated temperature due to climate change has negative consequences in reproduction and survival of ectotherms. Our results clearly indicate the importance of studying reproductive strategies from a life-history perspective to understand both developmental and transgenerational effects of temperature. Female strategy for allocating energy and resources among consecutive reproductive events may be an adaptive mechanism mediating transgenerational phenotypic plasticity for preparing offspring to grow and survive better in the unfavourable environment. However, our results cannot explicitly demonstrate whether the temperature-induced changes in maternal strategy are due to constraints imposed by higher energetic costs for self-maintenance of the mother or an adaptation. Future studies are needed to test whether this female strategy is indeed beneficial for offspring in warming environments.

## Additional files

**Additional file 1.** Tables S1–S4 and figures S1–S2.

**Additional file 2.** Data S1. Raw experimental data.

## Abbreviations

LME: linear mixed-effect
GLMM: generalized linear mixed model
CN: clutch number
NS: total number of spawning events
