# Supplementary material for: Thermal conditions during early life influence seasonal maternal strategies in the three-spined stickleback
Source: BMC Ecol. 2017 Nov 10;17:34. doi: 10.1186/s12898-017-0144-x (PMC5681783; doi:10.1186/s12898-017-0144-x)

Additional file 1: Tables S1-4 and Figures S1-2

Table S1 Linear mixed effect (LME) model of clutch mass including treatment (control or warm winter), individual-based clutch number and its quadratic term, number of spawnings and their interactions as fixed effects. Female identity nested within growth tank and family was included as random effects. Analyses were based on 1585 clutches from 291 females. Non-significant interactions were excluded in the presented model

Fixed effects	Estimate ± SE	df	F	p
Intercept	119.763 ± 5.393			
Treatment, warm	-9.251 ± 4.1708	1, 75	0.191	0.664
Clutch number (CN)	-1.832 ± 1.005	1, 1290	5.112	0.024
CN^2	-0.378 ± 0.092	1, 1290	8.887	0.003
Number of spawnings (NS)	0.275 ± 0.631	1, 182	7.486	0.007
Treatment × CN	2.226 ± 0.524	1, 1290	14.054	< 0.001
NS× CN	0.429 ± 0.122	1, 1290	12.431	< 0.001
**Random effects**	**Variance**	**Proportion**	χ^2_1	**p**
Identity	24.565	0.377	156.717	< 0.001
Growth tank	6.976	0.107	0.262	0.609
Family	7.542	0.116	1.207	0.272
Residual	26.024			

Table S2 Linear mixed effect (LME) model of clutch size including treatment (control or warm winter), individual-based clutch number, number of spawnings and significant interactions as fixed effects. Analyses were based on 1582 clutches from 290 females. Female identity nested within growth tank and family was included as random effects

Fixed effects	Estimate ± SE	df	F	p
Intercept	6.468 ± 0.144			
Treatment, warm	-0.260 ± 0.126	1, 75	0.260	0.612
Clutch number (CN)	-0.092 ± 0.010	1, 1290	77.431	< 0.001
Number of spawnings (NS)	0.021 ± 0.015	1, 181	2.061	0.153
Treatment × CN	0.056 ± 0.0154	1, 1290	13.286	< 0.001
Random effects	Variance	Proportion	χ^2_1	p
Identity	0.689	0.345	129.127	< 0.001
Growth tank	0.277	0.139	0.990	0.320
Family	0.247	0.124	1.346	0.246
Residual	0.787			

Table S3 LME model of egg mass based on 1581 clutches from 290 females

Fixed effects	Estimate ± SE	df	F	p
Intercept	2.685 ± 0.080			
Treatment, warm	0.013 ± 0.046	1, 75	0.018	0.892
Clutch number (CN)	0.090 ± 0.016	1, 1289	156.1	< 0.001
Number of spawnings (NS)	0.021 ± 0.008	1, 181	7.098	0.001
NS × CN	-0.003 ± 0.001	1,1289	5.467	0.019
**Random effects**	**Variance**	**Proportion**	χ^2_1	**p**
Identity	0.228	0.223	33.18	< 0.001
Growth tank	0.113	0.111	1.123	0.289
Family	0.194	0.190	8.489	0.004
Residual	0.487			

Table S4 LME model of egg carotenoid concentration based on 1358 clutches from 279 females

Variable	Estimate ± SE	df	F	p
Intercept	13.297 ± 0.473			
Treatment, warm	1.138 ± 0.513	1, 73	0.003	0.956
Clutch number (CN)	-0.181 ± 0.116	1, 1075	2.386	0.123
CN^2	0.012 ± 0.009	1, 1075	12.11	< 0.001
Number of spawnings (NS)	0.073 ± 0.043	1, 172	2.482	0.117
Treatment × CN	-0.529 ± 0.193	1, 1075	0.241	0.624
Treatment × CN^2	0.045 ± 0.016	1, 1075	7.420	0.007
**Random effects**	**Variance**	**Proportion**	χ^2_1	**p**
Identity	1.316	0.227	22.262	< 0.001
Growth tank	0.591	0.102	0.619	0.431
Family	0.941	0.163	5.608	0.018
Residual	2.938			

Fig. S1 Comparisons of key life-history traits of female three-spined sticklebacks between the control (n = 159 females) and warm winter (n = 141 females) groups. Mean ± SE **a** standard length at the onset of the breeding season (ln-transformed), **b** fecundity (total number of eggs produced during the breeding season, square root-transformed) and **c** total number of spawning events (square root-transformed)

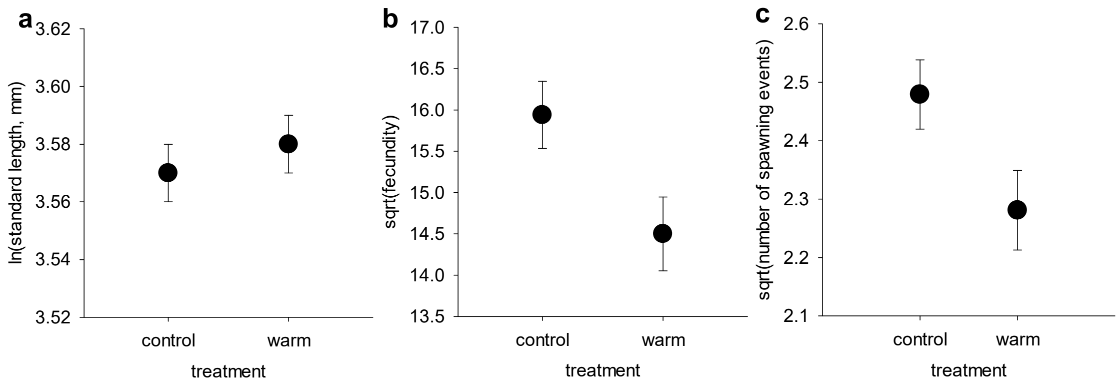

Fig. S2 Changes in **a** clutch mass and **b** egg mass according to clutch number with respect to number of spawnings during the season. For clarity of presentation the number of spawnings is categorised into three levels (red: 1-4 clutches, green: 5-8 clutches and blue: ≥ 9 clutches) but is treated as a continuous variable in all statistical analyses

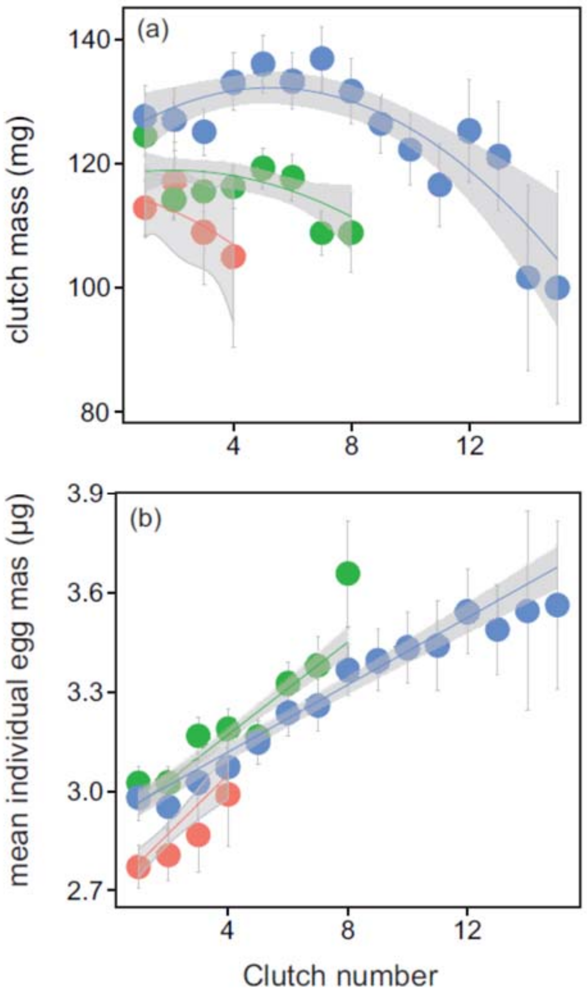

Supplement: Supplementary file 1 — Additional file 1. Tables S1–S4 and figures S1–S2. [file 12898_2017_144_MOESM1_ESM.pdf]
